# Sensitivity of Band-Pass Filtered In Situ Low-Earth Orbit and Ground-Based Ionosphere Observations to Lithosphere–Atmosphere–Ionosphere Coupling Over the Aegean Sea: Spectral Analysis of Two-Year Ionospheric Data Series

**DOI:** 10.3390/s24237795

**Published:** 2024-12-05

**Authors:** Wojciech Jarmołowski, Anna Belehaki, Paweł Wielgosz

**Affiliations:** 1 Faculty of Geoengineering, University of Warmia and Mazury in Olsztyn, Ul. Oczapowskiego 2, 10-719 Olsztyn, Poland; pawel.wielgosz@uwm.edu.pl; 2 Institute for Astronomy Astrophysics Space Applications and Remote Sensing, National Observatory of Athens, Metaxa & V. Pavlou, 15236 Penteli, Greece; belehaki@noa.gr

**Keywords:** Swarm, electron density, Digidonde, TEC, earthquake

## Abstract

This study demonstrates a rich complexity of the time–frequency ionospheric signal spectrum, dependent on the measurement type and platform. Different phenomena contributing to satellite-derived and ground-derived geophysical data that only selected signal bands can be potentially sensitive to seismicity over time, and they are applicable in lithosphere–atmosphere–ionosphere coupling (LAIC) studies. In this study, satellite-derived and ground-derived ionospheric observations are filtered by a Fourier-based band-pass filter, and an experimental selection of potentially sensitive frequency bands has been carried out. This work focuses on band-pass filtered ionospheric observations and seismic activity in the region of the Aegean Sea over a two-year time period (2020–2021), with particular focus on the entire system of tectonic plate junctions, which are suspected to be a potential source of ionospheric disturbances distributed over hundreds of kilometers. The temporal evolution of seismicity power in the Aegean region is represented by the record of earthquakes characterized by M ≥ 4.5, used for the estimation of cumulative seismic energy. The ionospheric response to LAIC is explored in three data types: short inspections of in situ electron density (Ne) over a tectonic plate boundary by Swarm satellites, stationary determination of three Ne density profile parameters by the Athens Digisonde station AT138 (maximum frequency of the F2 layer: foF2; maximum frequency of the sporadic E layer: foEs; and frequency spread: ff), and stationary measure of vertical total electron content (VTEC) interpolated from a UPC-IonSAT Quarter-of-an-hour time resolution Rapid Global ionospheric map (UQRG) near Athens. The spectrograms are made with the use of short-term Fourier transform (STFT). These frequency bands in the spectrograms, which show a notable coincidence with seismicity, are filtered out and compared to cumulative seismic energy in the Aegean Sea, to the geomagnetic Dst index, to sunspot number (SN), and to the solar radio flux (F10.7). In the case of Swarm, STFT allows for precise removal of long-wavelength Ne signals related to specific latitudes. The application of STFT to time series of ionospheric parameters from the Digisonde station and GIM VTEC is crucial in the removal of seasonal signals and strong diurnal and semi-diurnal signal components. The time series formed from experimentally selected wavebands of different ionospheric observations reveal a moderate but notable correlation with the seismic activity, higher than with any solar radiation parameter in 8 out of 12 cases. The correlation coefficient must be treated relatively and with caution here, as we have not determined the shift between seismic and ionospheric events, as this process requires more data. However, it can be observed from the spectrograms that some weak signals from selected frequencies are candidates to be related to seismic processes.

## 1. Introduction

The locations of the earthquakes are concentrated predominantly along tectonic plate boundaries, where small and large earthquakes occur frequently, assuring a quasi-constant process. The earthquakes result from tectonic processes, which, according to many recent studies, generate so-called lithosphere–atmosphere–ionosphere coupling (LAIC). The tectonic plate boundaries are weak places of the Earth’s lithosphere [1] and also places of various chemical and physical processes contributing to LAIC [2,3,4,5]. These are processes affecting the ionosphere from below, and their effects in the ionosphere are mixed with different processes from above, related to the sun and the geomagnetic field. The recent studies by Hayakawa et al. [2], Astafyeva et al. [6], and Afraimovich et al. [7] inform us about a suspected similarity of ionospheric responses to different types of earthquake sources and their tectonic settings and indicate a higher influence of various other factors on the ionospheric anomaly, such as the channel of wave propagation, the magnitude of the earthquake, or the scale of related tectonic displacements. The channel of LAIC is an important issue in this study. The effect of earthquake magnitude on the scale of ionospheric variations can also be observed in the presented analysis of ionospheric parameters. However, the exact epicenter locations, earthquake sources, and displacement influence must be analyzed in more specific local studies, where a different data preparation must be performed and separate spatiotemporal scales must be applied.

The focus on the tectonic plate boundary instead of the earthquake as an observational reference was not so popular but can be found in previous studies [8,9]. However, there are studies where the spatial reference is made with respect to some near zones, where ionospheric anomalies can be found [10,11,12,13]. An example of correlation analysis between high-energy electrons detected by POES satellites and earthquakes from a selected geographic sector in Asia can also be found in [14]. Therefore, spatial referencing of ionospheric anomalies to some near zones around the epicenter or just to a tectonic plate boundary is worth considering due to several reasons. First of all, the ionosphere starts around a hundred kilometers above the lithosphere, and the propagation of the electromagnetic anomalies from the lithosphere can diverge from near-vertical [15]. The upward propagation of an electric field anomaly can be affected by the atmospheric currents flowing into the ionosphere, and thus, the size of the disturbance can be different in comparison to that near the Earth. Secondly, in many cases, the horizontal data location can be only approximately linked with the lithospheric location. This applies to the case of global ionospheric maps (GIMs), which have only several hundred kilometers resolution, or to the case of satellite tracks, which pass close but not exactly over the earthquakes. The other cases of data available from the broad part of the ionosphere around the seismic events are Digisonde stations or GNSS stations. These stations can be placed at some distance from the epicenter, so the ionosphere that we observe is located at some horizontal distance from the earthquake. Finally, we know that only acoustic gravity waves (AGW) propagate during minutes to hours after the largest crustal movements, whereas electric field modifications (EFMs) can last for days or even tens of days [16], can be geographically wide [17], and, moreover, they can be precursory to the largest shocks [3,18,19]. Therefore, if we want to observe the variations related to an EFM channel, it is better to compare them to general seismic activity and other parameters over a longer time span instead of considering a short time around the single event. The studies of TEC by Parrot et al. [17] or Park et al. [20] proved that we can observe ionospheric anomalies distributed over hundreds of kilometers.

Many researchers focus on pre-seismic, co-seismic, and post-seismic ionospheric anomalies triggered by a selected single earthquake or several large events. These works show ionospheric anomalies detected in ground-based or satellite data close to the events and contribute substantially to LAIC explanation but will not be cited individually here, primarily due to their big number. Instead, for the review studies, grouping them very well can be indicated. There are discussions on LAIC components in [2,4,21,22,23]. There are also interesting, edited monograph series like Jin et al. [24], Ouzounov et al. [25], and Pulinets and Ouzounov [26], where one can find many references to individual literature examples of seismic-related ionospheric anomalies. These examples are not listed here also due to the second reason: the spatial shapes of ionospheric anomalies, their exact temporal correlation, and the links to selected earthquake events are out of the scope of this work. A more general study focused on the tectonic plate boundary is chosen to show how the ionosphere varies over a two-year period with respect to different strengths of seismic activity, as well as to various solar and geomagnetic conditions. The seismic activity is treated here similarly to, e.g., geomagnetic activity, as a quasi-continuous process, and some simple indicators of its intensity are estimated. Independent filtered two-year data series from Swarm satellites, Digisonde stations, and GIMs are investigated along the time axis with the application of spectral analysis. Therefore, the literature references in the next paragraphs are limited only to previously made spectral analyses of data from Swarm satellites, Digisonde stations, GIMs, and GNSS stations or at least to the analyses of time series with the application of other interesting methods, showing evidence of temporal electric field variations.

A Swarm satellite constellation includes three low-Earth orbit (LEO) satellites, which provide limited observations over a selected location daily but instead provide global coverage and reach all tectonic plate boundaries of the Earth. Swarm satellites fly over tectonic plate junctions at different longitudes on subsequent days, and due to the high speed of LEO, collect short data samples of electron density (Ne) observations over seismically active places. Swarm payload includes Langmuir Probes (LPs) and GNSS receivers for precise orbit determination, which can be used to observe in situ ionospheric Ne and topside TEC, respectively. The performance of LPs providing Ne for this analysis is described by Knudsen et al. [27] and Catapano et al. [28]. He et al. [29] have significantly extended the analyses of Swarm Ne disturbances referring to seismic activity. They have analyzed the global distribution of Swarm Ne disturbances and compared them to the areas of higher and lower seismic activity, rather than to specific earthquakes, which has a common point with our current study. Stanica et al. [10] applied magnetic and electric field observations from Swarm along-track measurements and applied spectral analysis to suspected precursory signals before a moderate-magnitude earthquake in Romania. The processing of obtaining LEO data from fast Swarm satellites substantially requires a spectral approach to distinguish transient anomalous signals in the complex spectrum of data derived from fast-moving satellites. Fortunately, the frequency domain was more commonly used in the last years in the processing of LEO data, and we can find principal component analysis of Swarm Ne and magnetic observations in [16], wavelet analysis of CHAMP magnetic data in [30], and wavelet analysis of Swarm magnetic data in [31]. Additionally, Du and Zhang [32] extended wavelet application to the analysis of ion densities measured by the China Seismo-Electromagnetic Satellite (CSES). The spectrograms based on the short-term Fourier transform (STFT) describing variable power spectral density (PSD) of magnetic data from Swarm can be found in [33]. The STFT is also applied to Ne data in [34,35] in the search of seismic-related anomalies observed by LPs along Swarm satellite tracks. A similar approach is applied in the current work to Ne data from Swarm, but more spectrograms are calculated, and PSD is sampled at a selected frequency, in order to prepare its time series for further analysis.

Ground GNSS receivers provide a large amount of data. The number of the receivers is large, but their distribution is inhomogeneous, which leaves several seismically active plate boundaries without ground GNSS data. The distribution of LEO orbits is more universal in this regard, although they have much lower temporal resolution over a specific location. The use of spectral analysis over station-based GNSS data in the search of ionospheric disturbances is the most frequent, probably also due to the wide data availability. There are examples of wavelets applied to several-hour ground GNSS observations in [36,37,38,39]. All these publications present wavelet scalograms of at most several hours of ground GNSS TEC and analyze sub-diurnal wave periods. Shorter-wave periods were analyzed in the spectrograms of several-hour series of ground-based TEC in [40,41,42,43,44]. Rolland et al. [45] proceeded even further, and starting from the spectrogram of TEC time series, they determined different spectral patterns for different types of disturbing waves using their speed with respect to GNSS station. In the current study, the method is also STFT, but TEC is interpolated from a UPC-IonSAT quarter-of-an-hour time resolution rapid global ionospheric map (UQRG GIM) instead of from the station data. The comparison of GIM with station data proved that analysis of the wave periods longer than semi-diurnal is well effective using UQRG with 15 min temporal resolution. Moreover, it was easier to generate two-year data series from GIM than from the station observations.

The ionospheric parameters determined by Digisonde stations and potentially sensitive to LAIC processes were also investigated using short-term data samples in [46,47,48,49]. It was also proven that seismic-driven ionospheric anomalies are in some cases detectable even in raw ionograms. Nevertheless, the characteristics of ionospheric electron density profiles determined from ionograms are more interesting, as their parameters can form data series representing their temporal variations. For the purposes of this investigation, we analyze three ionospheric characteristics: foF2, foEs, and ff. According to [50], the maximum frequency and the virtual height of the F2 layer at middle latitudes exhibit significant variations for several days prior to and after the earthquakes. The frequency spread, ff, is a characteristic that it is often connected with the presence of wave-like disturbances consistent with traveling ionosphere disturbances (TIDs) or AGWs, which, in turn, can be the effect of strong earthquakes (Alfonsi et al., 2024) [51]. Finally, sporadic E layers are examined regarding their response to seismogenic zonal electric fields, and this has been studied by Kim et al. [52] and by Xu et al. [53], who proposed a physical model of sporadic E layers based on wind shear theory at middle latitudes, resulting in a complicated response.

Temporal variations in ionospheric data series can be analyzed over several days, with respect to the earthquakes [54]. Koucká Knížová et al. [55] presented several-day samples of ionospheric profile characteristics spectrally analyzed with the use of wavelets. They found diurnal and several-day periodicities but also evidence of seismic-driven anomalies. Thus, data from Digisonde stations have a strong diurnal signal component resulting from the solar activity, and therefore spectral band-pass filtering is particularly appropriate in their preprocessing. Nevertheless, the use of spectral tools is less frequent in the analysis of Digisonde-based time series in comparison to GNSS TEC. An example can be found in Sonakia et al. [56], where they applied wavelets and scalograms in the analysis of the monthly record of critical F2 frequency (foF2) in order to reveal its sensitivity to earthquakes. The current work processes two-year series of three Digisonde parameters in exactly the same way as GIM-based TEC.

The objective of this study is to apply experimental STFT analysis of two-year series of Swarm in situ Ne, three characteristics from Athens Digisonde, foF2, foEs, and ff, and TEC interpolated from a UPC-IonSAT quarter-of-an-hour time resolution rapid global ionospheric map (UQRG) in the ionosphere over the junction of Eurasian and African tectonic plates. The first advantage of this work is that three types of data are used and compared. The second advantage is that two years of data are analyzed in this study, which provides a view of seasonal effects. The third and most important advantage is the application of spectral analysis and decomposition of all the used signals over the time span of two years. The spectral properties of ionospheric characteristics are analyzed with the use of discrete Fourier transform (DFT) and STFT. The approach is similar for GNSS-based and Digisonde-based parameters but slightly different for Swarm data. The Ne density profile parameters and TEC are continuous discrete signals in time and stationary with respect to location. UQRG GIM assured the time series of 15 min resolution, whereas the Digisonde provided the series of 5 min, and both data types are analyzed after filling the gaps with zero values with respect to the time unit by STFT. The STFT-based spectrograms assure indicative assessment of all frequency bands, and the visual inspection suggests the bounds of filtering. The analysis investigates wave periods longer than diurnal after filtering out diurnal and sub-diurnal signals. Selected signals are compared with seismic activity and geomagnetic and solar parameters. TEC time series interpolated from UQRG GIM are processed in the same way as Digisonde time series. In turn, to create time series from a Swarm Ne signal, some frequencies must be selected from along-track data. Therefore, STFT is applied to Swarm data at the stage of along-track data, and selected signal frequencies form time series for the further correlation analysis over two years of time. Summarizing, STFT is performed for each data type at the stage when the data are continuous and regular, and interesting wave periods potentially including seismic ionospheric responses are filtered out.

## 2. Methodology

### 2.1. Discrete Fourier Transform and Power Spectral Density

The discrete Fourier transform (DFT) is an approximation of the continuous Fourier transform. The continuous Fourier transform assumes an infinite signal, whereas in DFT, discrete real data are limited, i.e., the signal amplitude is zero beyond the end of the time series. The forward and inverse DFT are given in [57]:

$$X_{f}=\sum_{n=0}^{N-1}x_{n}W_{N}^{-fn}\;\;\;\;\;\;\;\;\;\;\;\;\;\;\;\;\;\;\;\;\;\;\;\;\;\;\;\;\;\;\;\;\;f=1,\;2,\ldots ,N-1$$

(1) $$x_{n}=\frac{1}{N}\sum_{f=0}^{N-1}X_{f}W_{N}^{fn}\;\;\;\;\;\;\;\;\;\;\;\;\;\;\;\;\;\;\;\;\;\;\;\;\;\;\;\;\;\;\;n=1,\;2,\ldots ,N-1,$$

where the complex quantity *W_N_*, known as the twiddle factor, is defined by $W_{N}=e^{\frac{i2\pi }{N}}$. The vector $x_{n}$ is a sequence of discretized time–domain signals to be transformed, f is the harmonic number, n is the number of samples, and *N* is the number of all samples. The sampling interval is here Δ*t* = *T*/*N*, for the line spectrum at frequency $\omega_{f}=\left(2\pi \right)\frac{f}{T}$, and the transform has therefore a limited frequency resolution $2\pi \frac{1}{T}$. The signal of time function *x*(*t*) can be decomposed into frequency responses *X*(*ω*) sampled for the entire ω spectrum. These responses can be also transformed back exactly to *x*(*t*) by the inverse Fourier transform. The selected signal band can be subtracted from the data, which, contrary to simple methods of detrending, e.g., moving average or polynomial, removes exactly this part of the signal that we want to remove in terms of a frequency band. The discrete autocorrelation function of *x_n_* is:

(2) $$R_{xx}=\frac{1}{N}\sum_{n=0}^{N-1}x_{n}x_{n-l}\;\;\;\;\;\;\;\;\;\;\;\;\;\;\;\;\;\;\;\;\;\;\;\;\;\;\;\;\;\;\;\;\;l=1,2,\ldots ,N-1$$

The Fourier transform of the autocorrelation function for the sample $x_{n}$ is defined as the power spectral density (PSD):

(3) $${PSD}_{xx,f}=\sum_{n=0}^{N-1}R_{xx}W_{N}^{-fn}\;\;\;\;\;\;\;\;\;\;\;\;\;\;\;\;\;\;\;\;\;\;\;\;\;\;\;\;\;\;\;\;\;f=1,2,\ldots ,N-1$$

The amplitude of signal responses in the frequency domain provides PSD at individual frequencies/wavelengths. It is typical that three frequency domain units can be applied in the terminology alternately, i.e., frequency, wavelength, as well as wave period.

### 2.2. Short-Term Fourier Transform and Evolutionary Spectral Density

The PSD can be calculated for the entire length of the analyzed time series, producing one-dimensional power spectra at different frequencies. The STFT enables the calculation of time-variable PSD [58]. The evolutionary and windowed STFT is a modification of the DFT method, which computes the spectrum of overlapping segments of the time series. The data sequence to be transformed is multiplied by a window function which is nonzero for a short period of time [58,59]. The STFT of the signal is computed as the window is sliding along the time axis, resulting in a two-dimensional representation of the signal. Mathematically, this is written as:

(4) $${STFT}_{x,f,t}=\sum_{n=0}^{N-1}x_{n}w_{n-t}W_{N}^{-fn}\;\;\;\;\;\;\;\;\;\;\;\;\;\;\;\;\;\;\;f=1,2,\ldots ,N-1\;$$

where $w_{n}$ is a discretized window function and t is its time index. STFT of a data sequence $x_{n}$ describes its local spectral content near the moment t as a function of f. Moving the center of the window $w_{n-t}$ along the time axis allows for obtaining snapshots of time–frequency behavior of $x_{n}$. STFT is used in the calculation of PSD for each sample. These PSDs form then a 2D spectrogram shape, which depends on the window size and windows’ overlay size. This corresponds to the computation of the STFT squared magnitude of the signal sequence x_n_:

(5) $$S_{xx,f,t}={\left|{STFT}_{x,f,t}\right|}^{2}$$

STFT, as a windowed Fourier transform, is a very flexible approach in terms of parametrization, band-pass filtering, and its adjustment to the required resolutions. The size and shape of the analysis window can be modified depending on the purpose. A shorter window will produce more accurate results in timing at the expense of precision of frequency representation. A longer window will provide a more precise frequency representation at the expense of precision in time location.

## 3. Data Selection and Preprocessing

### 3.1. Seismic Data Record

**Figure 1:**
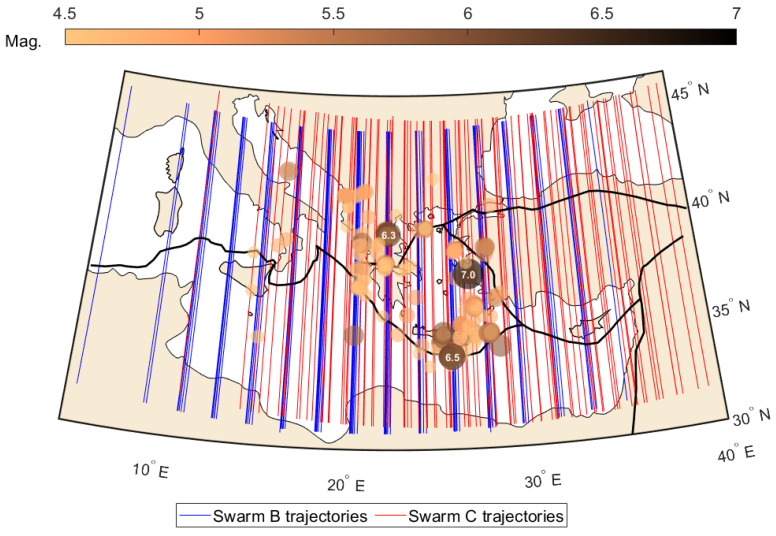
Selection of Swarm B and C tracks in the last quarter of 2020 together with the epicenter of earthquakes that occurred at that time in the Aegean Sea and neighboring regions. The tectonic plate boundaries are also presented in this map.

The magnitudes of the earthquakes in 2020–2021 reached 7.0, and these years experienced the strongest seismic activity in the region of the Aegean Sea during the Swarm mission. However, this two-year time period includes also relatively quiet time spans, which are also discussed. The studies are based on the data from the area of Aegean Sea, collected over two years (2020–2021), which included the time of the M = 7.0 earthquake in October 2020. This event happened close to the Samos Island, but several other groups of earthquakes exceeding M = 4.5 happened in different parts of the Aegean Sea in the years 2020–2021 and were analyzed in this work. There were three–four such groups annually, and the seasons of their occurrence were different within these two years, which eliminates the suspicion of seasonal effects. The small depth of the analyzed earthquakes was important and probably contributed to the finding of interesting Swarm signal disturbances potentially related to seismically induced ionospheric disturbances. However, we provide depths in the figures to retain more information and to indicate infrequent deeper earthquakes having potentially a smaller contribution to cumulative seismic energy. The two-year time period is analyzed in this study, and such extended time was selected due to initially observed long-term behavior of Swarm signal disturbances, which turned out to be more long term than expected [60,61]. The earthquakes from M ≥ 4.5 were selected within a geographical range of 33° N to 43° N and 10° E to 30° E. The investigated earthquakes were located in the Aegean Sea area and surrounding countries, i.e., Greece, Turkey, Albania, North Macedonia, Bulgaria, etc. The sequences of seismic events are extracted from the United States Geological Survey (USGS) seismic record database, and the information of primary interest is their time, location, magnitude, and depth. The selection is limited to close vicinity of the Hellenic Tectonic Plate, which is located in the center of the most active part of the Eurasian/African plate boundary (Figure 1).

### 3.2. Swarm B and C Data

The applied Ne originates from the L1b level, and it is stored in EFI_LP files. The data from Swarm satellites are Ne values measured in situ at altitudes around 460 km (Swarm A/C) and around 520 km (Swarm B) during fast run of the spacecraft with speed equal to 7.6 km/s. These altitudes are slightly above ionospheric peak height and therefore operate in the F region and conditions of relatively high ionization sensitive to seismicity [29]. The fast orbital movement causes that detected along-track Ne irregularities are rather referred to as spatial variations of the ionosphere than as temporal ones, and therefore we decided to analyze yearly Swarm data spans to review temporal variations. Additionally, the fast run is suspected to contribute to the complexity of the signal in terms of frequency, which means that irregularities occupy a wide frequency band. The advantage of LEO satellites is their spatial access to locations where the installation and operation of ground-based GNSS receivers and ionosonde stations is not possible, such as the sea and oceans. The orbital tracks of LEO can make short inspections in all seismically active zones of the world. The drawback of LEO is that the satellite data are dense only along the orbital tracks. The one-dimensional nature of LEO along-track Ne observations, and a limited chance for spatiotemporal correlation due to the non-repeating orbits, strongly requires spectral analysis for a better recognition of disturbing signals. Among the three Swarm satellites, the tracks of Swarm B and C that fly over the seismically active Aegean Sea have been selected, because the data from Swarm A were incomplete. On the other hand, the observations from A and C are similar to a large extent, as was reported in a previous paper [35]. Depending on the phase of the Swarm constellation, consecutive single satellite orbital tracks occur at approximate longitudes at least two times per day, which results in one track closer to noon time, and a second one closer to midnight. The nocturnal observations are theoretically less affected by the solar activity. Therefore, in this analysis, as a first choice, nighttime tracks are selected. Nevertheless, sometimes the observations refer to the late evening or early morning, but this extension of time is inevitable if we need to include in our sample at least one Swarm pass every day of the year, and close to the Hellenic Plate. Additionally, in order to keep continuity of the observations, the longitudes of selected tracks must vary. However, the active Eurasian/African plate boundary has a favorably significant length and longitudinal direction, and this orientation assures an almost perpendicular pass of the Swarm satellite.

The satellite tracks are grouped into four quarters of the year (2020 and 2021). Firstly, a reference track is selected close to the center of the region. Typically, this Swarm pass takes place in the middle of the quarter (day 45 or 46), but there are some exceptions, and occasionally the reference track is selected on a day distant from the middle of the selected time period (e.g., 65th). This is necessary in order to find a Swarm pass possibly close to the center of the region and in the nighttime. Once such reference track is selected, then around 90 tracks (one pass daily) are adjoined, with a longitudinal separation from the reference track smaller than 20° and a temporal separation in UTC time smaller than 4.6 h. These apparently large thresholds are required to ensure that observations over the region correspond to approximately the same time and location and also to ensure tracks are considered almost daily, having only minor single-day gaps. Summarizing, two years of Swarm passes over the boundary between the African and Eurasian tectonic plates (in the close vicinity of the Hellenic Plate) have been selected—one track daily during the nighttime. The selection has minor gaps due to variable longitudes of Swarm orbit footprint, but these gaps do not affect overall findings. The example selection of Swarm B/C trajectories in the last quarter of 2020 is shown in Figure 1, together with selected earthquakes at that time. The selections in the other quarters of 2020 and 2021 follow the same rules, and respective locations of the tracks depend only on the current Swarm constellation geometry. The longitudinal location of the tracks can differ by 40°, but all the passes cross the tectonic plate connection orthogonally, which is an important assumption in this study.

**Figure 2:**
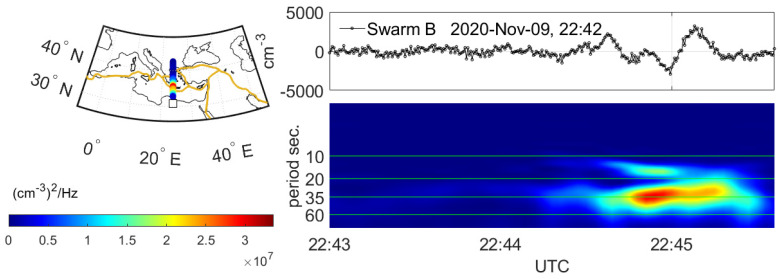
Example residual Swarm Ne data (**upper right**), example spectrogram of suspected co-seismic Ne disturbance detected by Swarm B (**lower right**), and Swarm PSD sampled at 35 s wave period with tectonic plate boundaries (**left**).

Swarm satellite data are dense only along the orbital tracks, and ionospheric disturbances can be observed in fact in one dimension, which is along the track. However, the spectral analysis facilitates a better insight into the one-dimensional nature of Swarm along-track observations. Therefore, Swarm along-track Ne data from LPs are analyzed by one-dimensional STFT. Prior to this, the Ne signal is high-pass filtered by a DFT-based filter. The lower bound of filtering was set to 50 s (~380 km), which assured elimination of different natural spatial Ne variations like equatorial ionization anomaly (EIA) crest. The STFT analysis and the spectrograms of Ne along-track data have shown that a wide band of Ne signal wave periods are potentially sensitive to seismically driven disturbances. It is noticeable in the example spectrogram (Figure 2) that the disturbances occupy a wide range of wavelengths, and it is hard to find a very narrow frequency band that is solely affected by detected Ne perturbations potentially suspected of co-seismic origin. Therefore, selection of one specific sensitive frequency can be only approximately performed at this stage of the research. In this work, we choose a wave period of 35 s, which is equivalent to around 270 km wavelength, because it appears as sensitive to increased seismic activity in most cases. The PSD at this frequency is selected to characterize the power of disturbances detected by Swarm satellites and to compose its two-year series for the temporal analysis of Swarm Ne sensitivity to variable seismic activity.

### 3.3. Ground-Based Ionospheric Data—Athens Digisonde Station

**Figure 3:**
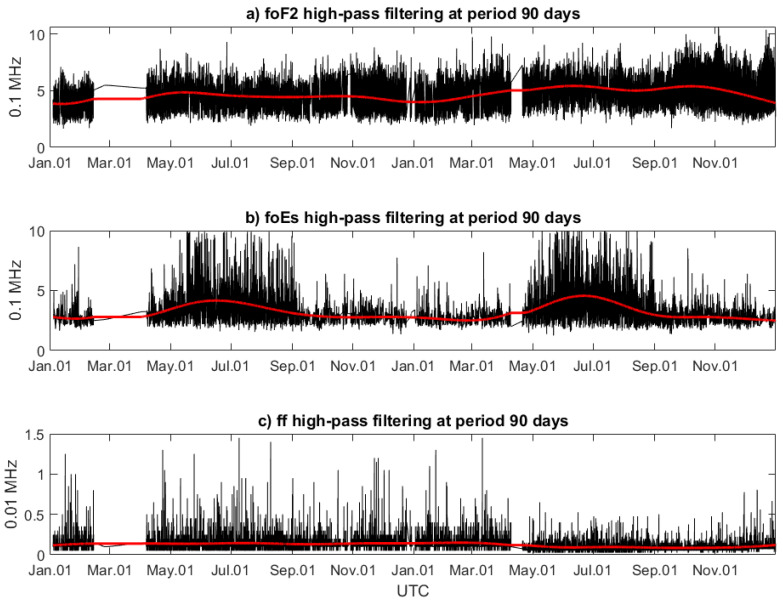
Critical frequency of (**a**) F2 layer (foF2), (**b**) sporadic E layer (foEs), and (**c**) spread frequency (ff) from Athens Digisonde (black) and their 90-day trends estimated by DFT (red) in 2020/2021. Data gaps are also ignored in further correlation analysis.

The ionospheric parameters determined by Digisonde are based on bottom-side measurements and, therefore, also reach the highly ionized F region, i.e., the region just below Swarm orbits. The altitudes of Digisonde measurements are closer to the lithosphere, and moreover, evidence of co-seismic ionospheric irregularities in Digisonde soundings was also reported in [48]. Three parameters are selected for STFT analysis: foF2 related to the highly ionized region just below Swarm altitudes, foEs potentially sensible to electric fields, which can create the anomalous Es layers, and ff potentially related to the turbulent ionosphere. It is worth mentioning that ionospheric variations related to earthquakes or their precursors in the F region and Es layer were studied several times, and similar parameters were applied [62]. The two-year time series of three parameters, foF2, foEs, and ff, were derived from the Athens Digisonde observations. These time series have a significantly complex frequency spectrum. Some of these frequency bands, and, in particular, those dependent on solar conditions, adversely affect the recognition of sensitivity of ionospheric parameters to the seismic activity. The main unwanted effects come from the seasonal influence of the Sun, as well as from diurnal variation of the solar radiation. The former effect occupies several-week wave periods in the analyzed data series but also several-day ones. The latter effects occupy higher, diurnal and semi-diurnal frequencies. Therefore, the signal has to be band-filtered to be useful in the analysis of Earth-dependent ionospheric disturbances coupled with internal lithospheric activity. The seasonal low-frequency signal, which is to be removed, is very clearly noticeable in the foEs parameter (Figure 3b).

The assessment of signal components of ionospheric Ne density profile parameters is performed with the use of STFT and the spectrograms. However, Fourier analysis requires continuous data series and therefore the gaps in the observations have to be filled. This is performed with simple linear interpolation, because such kind of the interpolation generates the minimum amount of artificial signals at higher frequencies. More precisely, linear interpolation is quite safe because we cannot generate frequencies shorter than the gap size except for at the bound equal to the size of the gap. Such artificial signal is therefore easy to be removed and is well recognizable in the spectrogram. After filling the series, the data are high-pass filtered, and the 90-day trend is removed (Figure 3).

### 3.4. Ground-Based VTEC Data—UQRG Model near Athens

**Figure 4:**
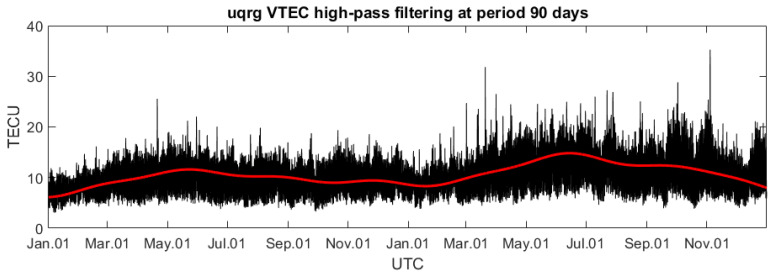
The VTEC interpolated from UQRG GIM near Athens (38° N and 24° E) (black) and its 90-day trend (red) in 2020/2021.

The ground-based VTEC is integrated over the spatial baseline including Swarm and Digisonde areas of detection and reaches even further to 20,000 km. This supplies more signal components, and therefore the VTEC signal is the most complex one out of the selected three. This complexity makes the spectral analysis of such signal more challenging, and it requires many iterations and processing with modified parameters. Therefore, at this experimental stage, we can determine the most obstructing signals, and at the same time, the most unwanted wave periods in the detection of co-seismic disturbances in the ionosphere. Similarly to Digisonde data, the same lower bound of the high-pass filter of 90 days is applied to VTEC data (Figure 4). This long-wavelength trend removal facilitates the analysis of shorter wavelengths. Two years of observations provide a good sample for the analysis of periodic signals within the range of 1–90 days. However, the determination of VTEC between the receiver and single GNSS satellite is possible only over several hours. Therefore, the generation of two-year data series from, e.g., the NOA1 GNSS receiver, would require averaging of VTEC observations to several satellites at different elevations or even need inclusion of more stations for VTEC calculation and averaging of VTEC over some area surrounding Athens. This would be an interpolation, being more simplified than stochastic (kriging) modeling, applied in the creation of UQRG GIM. Moreover, the shortest analyzed wave period here is the diurnal one, which does need high temporal resolution of GNSS data series. Therefore, it is decided to use VTEC interpolated from GIM at this stage of the research. The UQRG provides a model computed by tomography and kriging modeling [63,64]. Presently, UQRG is the most or one of the most accurate GIMs [65,66,67]. These global VTEC maps give a unique view of the dynamics of the electron content at a global scale, with VTEC maps provided every 15 min, with a latency of 1 day. The horizontal grid resolution is 2.5° N-S and 5° E-W, which is the standard established for GIMs.

The two-year series of VTEC variations are linearly interpolated from UQRG GIMs at a fixed position of 38° N and 24° E, which is the approximate position for Athens’ location. Some single gaps resulting from no map availability are filled by the previous day’s values, with no interpolation to avoid artificial signal generation. Such two-year data series are then high-pass filtered at a lower bound of 90 days. The VTEC signal, similarly to Ne density profile parameters from the Digisonde, includes a strong diurnal and semi-diurnal component. These components have the highest amplitudes in the signal in Figure 4 and will be easily noticeable in the spectrograms in the next sections.

## 4. Variations of Band-Pass Filtered Ionospheric Parameters over a Two-Year Time Span

### 4.1. Swarm Ne Variations

The approximate selection of the 35 s (~270 km) wave period in Swarm along-track Ne data was based on the review of calculated spectrograms, where the chosen wave period appeared to be one of the most strongly occupied by disturbances during large earthquakes and seismic sequences. It must be pointed out that in the case of Swarm along-track signal, a wide frequency band is typically occupied by various occurring disturbances. The 35 s sampling wave period was selected as a preliminary compromise and applied to calculate the maximum PSD of Ne at this frequency, which is highly probable to be affected by LAIC at Swarm height. Moreover, these PSD maxima have shown a between-day longer-term correlation that does not coincide with individual earthquakes but often coincides well with overall increased seismicity in the region. It is therefore probable that some other LAIC processes aside from AGW also disturb these signals. Fan et al. [16] also have shown the evolution of Ne and magnetic anomalies from Swarm over tens of days, linked it with the preparation process before a large earthquake, and pointed to electrochemical coupling as the suspected main propagation channel of LAIC for longer-term disturbances. Therefore, in this study, Swarm PSD maxima are averaged using a 20-day window to analyze longer-term effects. Additionally, due to the same reason, a kind of simplified seismicity indicator was calculated, as based on 20-day averaging of the ratio between the magnitudes and depths.

**Figure 5:**
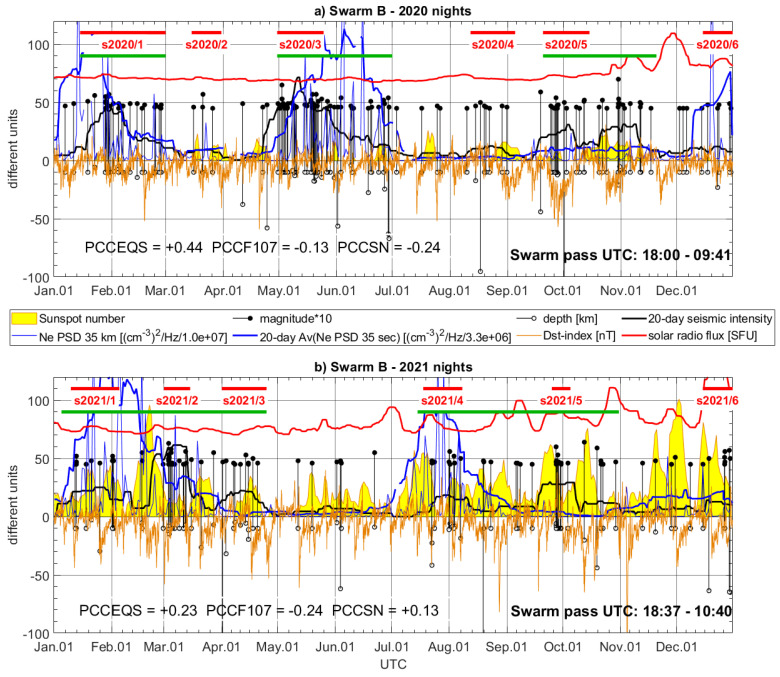
Maxima of PSD of Swarm B Ne disturbances in (**a**) 2020 and (**b**) 2021 (arbitrary scaling, blue narrow line) together with earthquakes in the Aegean region (magnitude multiplied by 10—black stems with dots, depth—black stems with circles). Max PSD have calculated the 20-day moving average (blue bold line). The earthquakes have calculated an indicator of seismicity (black bold line). The Dst index is plotted as an orange line. The sunspot number is represented by a yellow area plot. The solar radio flux is shown as a red line. The time periods indicated with red horizontal lines cover earthquake groups presented geographically in Figure 7. Green horizontal lines denote periods of higher seismicity.

**Figure 6:**
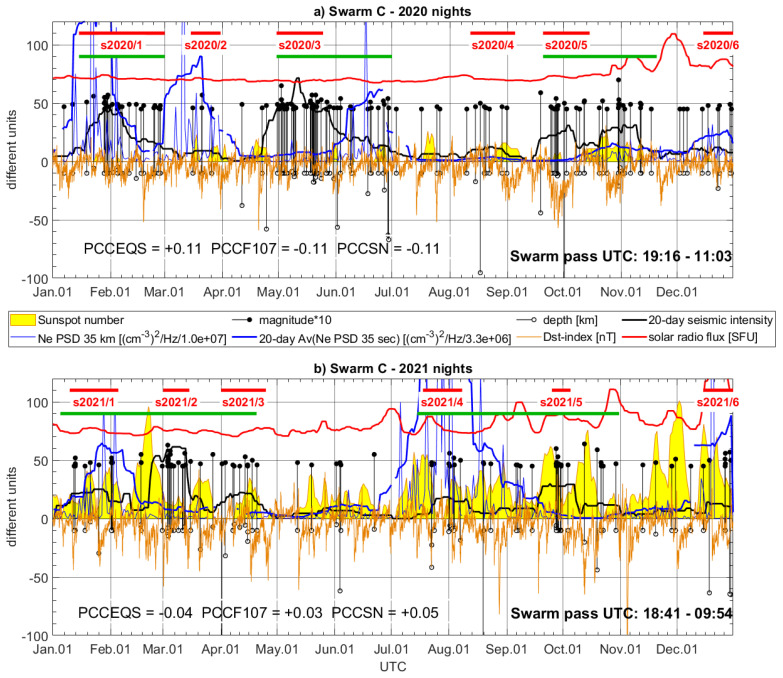
Maxima of PSD of Swarm C Ne disturbances in (**a**) 2020 and (**b**) 2021 (arbitrary scaling, blue narrow line) together with earthquakes in the Aegean region (magnitude multiplied by 10—black stems with dots, depth—black stems with circles). Max PSD have calculated the 20-day moving average (blue bold line). The earthquakes have calculated an indicator of seismicity (black bold line). The Dst index is plotted as an orange line. The sunspot number is represented by a yellow area plot. The solar radio flux is shown as a red line. The time periods indicated with red horizontal lines cover earthquake groups presented geographically in Figure 8. Green horizontal lines denote periods of higher seismicity.

The selected two-year data sample includes various seismic events, and also relatively quiet intervals, which constitutes a good test of sensitivity for Swarm LP measurements. The selected nocturnal Swarm track is represented every day by maximum PSD at 35 s. The temporal evolution of PSD maxima is presented separately for Swarm B and C and for two subsequent years in Figure 5 and Figure 6 (thin blue lines), together with their 20-day moving averages, which better reveal the longer-term character of the disturbances (bold blue lines). This averaging is performed after preliminary preview of PSD maxima, and the conclusion is that the temporal evolution of Ne disturbances is dynamic, and not so directly related to the largest earthquakes, but rather sensitive to earthquake series and different LAIC processes. The earthquakes in Figure 5 and Figure 6 are represented by their magnitudes multiplied by 10, to draw them together in the same scale with their depths, Dst index, sunspot number (SN), and solar radio flux (F10.7). Their depths are expressed in km by the stem plot directed to the bottom of Figure 5 and Figure 6. Based on earthquake magnitude and depth, and knowing that the shallowest earthquakes are the most suspected as affecting the ionosphere [11], we calculated a kind of seismic intensity indicator by the use of a simple rule of a 20-day window. The magnitudes of earthquakes occurring within this window are divided by their depths and then summed up and divided by the number of the days. The quantity calculated this way better reveals a long-term variation of seismicity, and, similarly to the moving average referred to as Swarm PSD maxima, it is drawn using bold lines of the same color as the input quantity, i.e., black bold line. The 20-day averages (of Swarm Ne PSD and earthquakes) can be more efficient in the visual analysis of the correlation of their two input quantities. Additionally, to assess potential influences and correlations of geomagnetic field and solar activity with Swarm Ne, the geomagnetic Dst index and F10.7 were added to Figure 5 and Figure 6. The external Ne disturbances, like the solar radiation or the geomagnetic activity, can be often mixed with those seismically triggered.

Even a rough look at bold black lines approximating the power of the earthquakes having M ≥ 4.5 enables a division of the year into shorter time periods of increased or decreased seismic activity. Seismically stronger time periods in the Aegean Sea occur two–three times annually. These roughly selected time periods of increase are indicated by green bold lines in Figure 5 and Figure 6. Figure 5a and Figure 6a show that in 2020, we can roughly indicate three time spans of highest seismic activity: 15 January–1 March; 1 May–31 June; and 20 September–20 November. When looking at year 2021 (Figure 5b and Figure 6b), we can find more shorter increases and decreases. Combining the most dense, we can distinguish two groups of stronger activity in the region: 05 January–25 April; and 15 July–31 October. Referring now back to 2020, we can say that the first two of the three time periods listed above coincide with the strongest disturbances of both Swarm B and C signals in 2020, which are observed during most of the increased seismicity period (Figure 5a and Figure 6a). The third period of seismicity enhance in 2020 is affected by moderate but also noticeable Ne perturbations (Figure 5a and Figure 6a). In 2021, a year when two longer time periods of stronger activity occurred, two intervals of intense Swarm B and C disturbances are observed (Figure 5b and Figure 6b). The first occurred from January to March for Swarm B (Figure 5b) and is shorter (January–February) for Swarm C (Figure 6b). The second group of strong and long-lasting disturbances starts at the beginning of July and is similarly long for both LEOs. We should also note that solar radio flux is low at least during the entire two first time periods in 2020 (Figure 5a and Figure 6a). The Dst index varies in 2020, but it can be noticed that at least for the second period, its value shows no extrema (Figure 5a and Figure 6a, May–June). The solar radio flux varies more in 2021, but the highest values are observed, as well as in 2020, at the end of the year, after the strongest earthquake series (Figure 5b and Figure 6b). The Dst index indicates a weak to moderate geomagnetic activity in both years.

The red horizontal lines in Figure 5 and Figure 6 indicate selected interesting shorter parts of earthquake groups. Figure 5a and Figure 6a indicate six shorter time periods in 2020. These groups can be helpful in the discussion on the size of disturbances in Swarm signals. An earthquake activity starts at the beginning of 2020 and intensifies towards the end of January (s2020/1), which can potentially have some foreshocks at the beginning of January. Swarm Ne disturbances are observed in the middle of January and can be potentially precursory signals to the strongest seismic activity at the end of January. In February 2020, the seismic activity remains increased, and Swarm B and perturbations decrease moderately. The second selected group of several relatively sparse earthquakes is denoted as s2020/2 in Figure 5a and Figure 6a. This is a moderate seismic activity increase, which is, however, preceded by one–two weeks of calm time relative to the M = 4.5 threshold. The group s2020/2 coincides with a moderate PSD increase in Swarm B Ne (Figure 5a) and rapid precursory increase in Ne PSD for Swarm C (Figure 6a). The time period s2020/3 covers two very intense earthquake series, one after another, which constitute the largest seismicity increase in 2020. The response of the ionosphere measured by Swarm B was immediate and strong (Figure 5a), whereas a strong rise in the case of Swarm C occurred at the end of this active interval, when some less frequent series of earthquakes appeared after the strongest ones (Figure 6a). The time interval s2020/4 includes sparse series of light earthquakes, which generate moderate seismic activity (similarly to s2020/2). Both Swarm satellites sense also this moderate activity increase as only a slight increase in Ne PSD. The last very dense (in time) group of earthquakes starts in the period s2020/5, together with an extreme Dst index. Swarm B observes disturbed Ne at that time. The increased PSD, although smaller than, e.g., in the case of s2020/3, persists until the largest M = 7.0 earthquake is located at the boundary of the Aegean Sea Plate (Hellenic Plate) and Anatolia Plate (location can be viewed in Figure 1). The increase in Swarm C PSD starts later but also before the strongest M = 7.0 earthquake (Figure 6a). The last significant PSD maxima increase coincide for both Swarm satellites at the end of the year (s2020/6), where a moderate seismicity increase is observed. The group s2020/6 has similar characteristics to group s2020/4, but it is also worth noting that s2020/6 happened after a large F10.7 increase.

**Figure 7:**
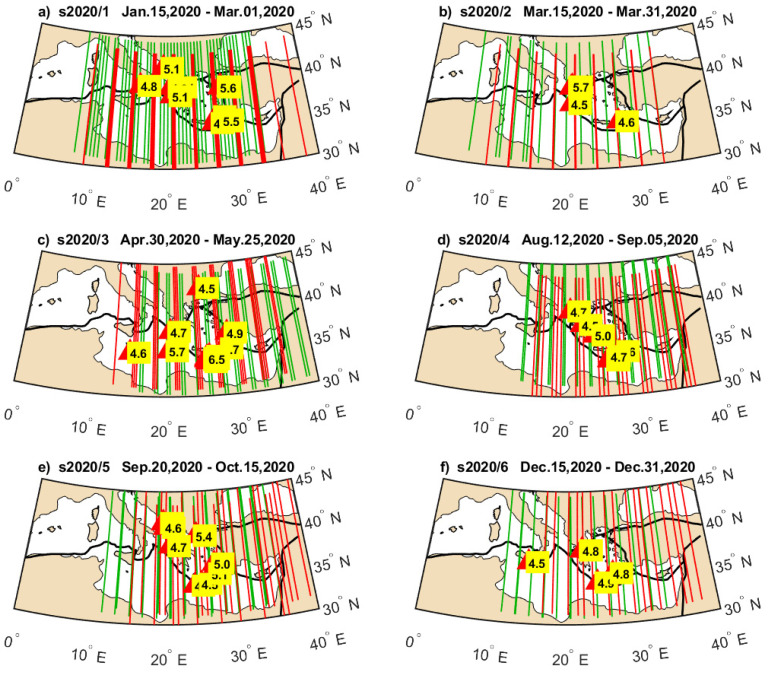
Geographical location of earthquakes occurring in selected periods in 2020 ((**a**–**f**) present earthquake groups indicated in Figure 5a and Figure 6a by red horizontal lines), together with Swarm B (green) and Swarm C (red) tracks at the same time.

**Figure 8:**
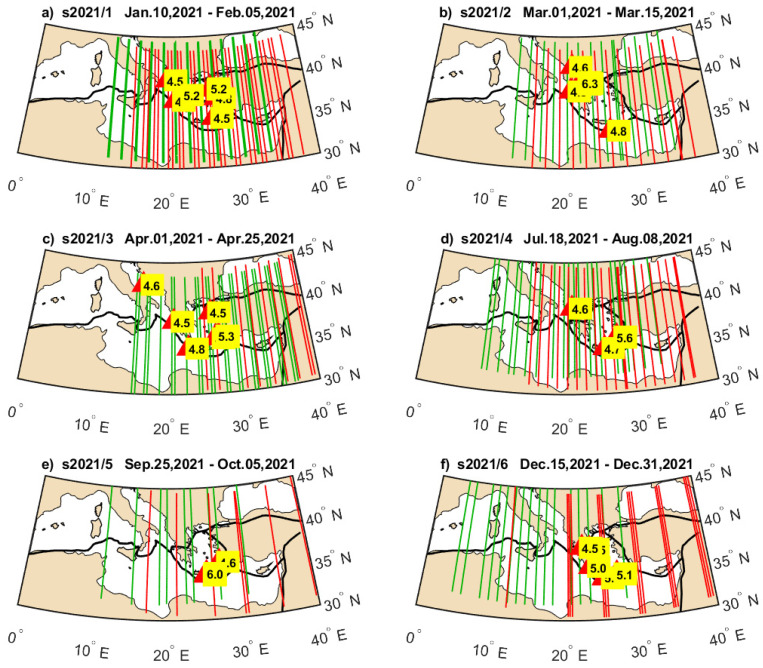
Geographical location of earthquakes occurring in selected periods in 2021 ((**a**–**f**) present earthquake groups indicated by red horizontal lines in Figure 5b and Figure 6b), together with Swarm B (green) and Swarm C (red) tracks at the same time.

**Figure 9:**
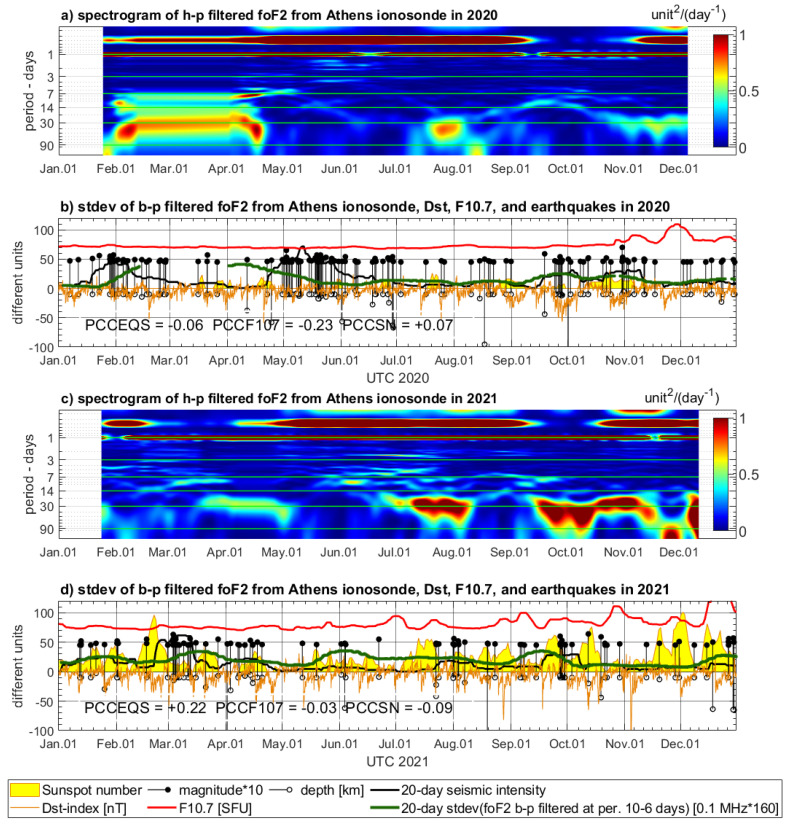
STFT analysis and band-pass filtering of foF2 parameter from Athens Digisonde in 2020 (**a**,**b**) and in 2021 (**c**,**d**). Subfigures (**a**,**c**) are spectrograms of high-pass filtered (90 days) signal, whereas (**b**,**d**) show standard deviation of band-pass filtered signal (10–6 days) calculated using a 20-day window. The foF2 is compared to the Dst index (orange), sunspot number (yellow area), solar radio flux (red), and magnitudes and depths of the earthquakes occurring in the Aegean region (magnitude multiplied by 10—black stems with dots, depth—black stems with circles). The earthquakes have a calculated indicator of seismicity (black bold line).

**Figure 10:**
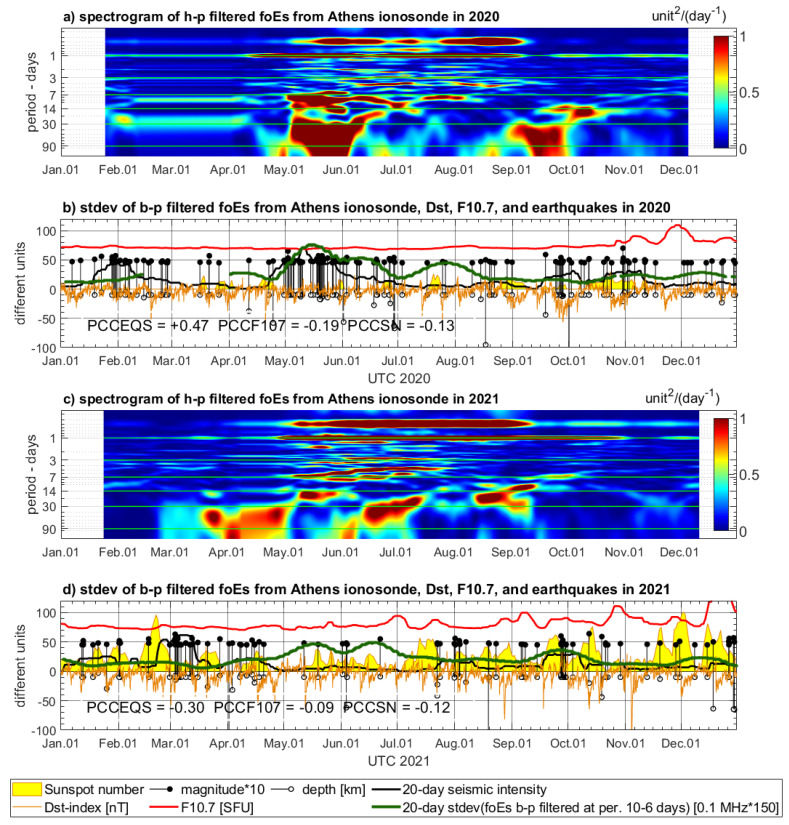
STFT analysis and band-pass filtering of foEs parameter from Athens Digisonde in 2020 (**a**,**b**) and in 2021 (**c**,**d**). Subfigures (**a**,**c**) are spectrograms of high-pass filtered (90 days) signal, whereas (**b**,**d**) show standard deviation of band-pass filtered signal (10–6 days) calculated using a 20-day window. The foEs is compared to the Dst index (orange), sunspot number (yellow area), solar radio flux (red), and magnitudes and depths of the earthquakes occurring in the Aegean region (magnitude multiplied by 10—black stems with dots, depth—black stems with circles). The earthquakes have a calculated indicator of seismicity (black bold line).

**Figure 11:**
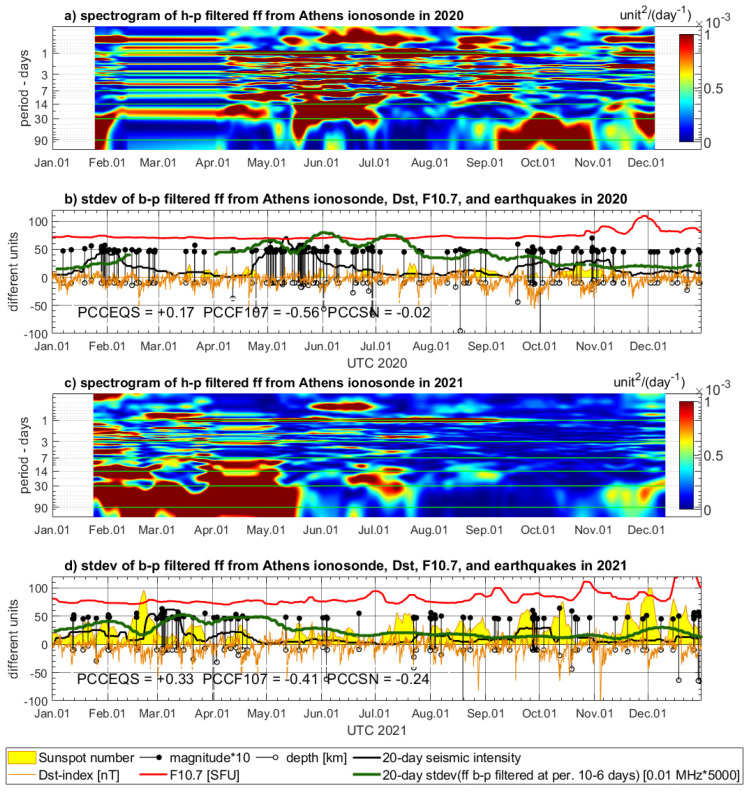
STFT analysis and band-pass filtering of ff parameter from Athens Digisonde in 2020 (**a**,**b**) and in 2021 (**c**,**d**). Subfigures (**a**,**c**) are spectrograms of high-pass filtered (90 days) signal, whereas (**b**,**d**) show standard deviation of band-pass filtered signal (6–10 days) calculated using a 20-day window. The ff is compared to the Dst index (orange), sunspot number (yellow area), solar radio flux (red), and magnitudes and depths of the earthquakes occurring in the Aegean region (magnitude multiplied by 10—black stems with dots, depth—black stems with circles). The earthquakes have a calculated indicator of seismicity (black bold line).

**Figure 12:**
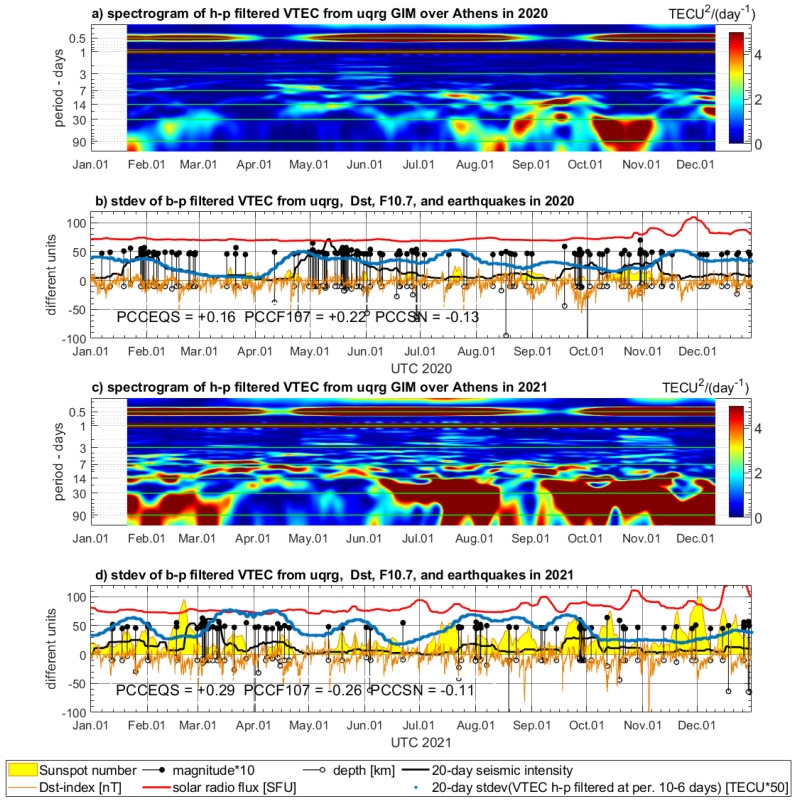
STFT analysis and band-pass filtering of VTEC interpolated near Athens from UQRG in 2020 (**a**,**b**) and in 2021 (**c**,**d**). Subfigures (**a**,**c**) are spectrograms of high-pass filtered (90 days) signal, whereas (**b**,**d**) show standard deviation of band-pass filtered signal (10–6 days) calculated using a 20-day window. VTEC is compared to the Dst index (orange), sunspot number (yellow area), solar radio flux (red), and magnitudes and depths of the earthquakes occurring in the Aegean region (magnitude multiplied by 10—black stems with dots, depth—black stems with circles). The earthquakes have a calculated indicator of seismicity (black bold line).

A notable visual correlation of Swarm Ne disturbances with seismic activity also encouraged supplementary calculation of Pearson correlation coefficients (PCCs) between averaged Swarm and seismicity denoted in Figure 5, Figure 6, Figure 7, Figure 8, Figure 9, Figure 10, Figure 11 and Figure 12 as PCCEQS, Swarm, and sunspot number denoted as PCCSN and Swarm, with solar radio flux denoted as PCCF107. The averaged Swarm Ne PSD, as well as 20-day cumulative seismic intensity, is applied for PCC calculation. The F10.7 parameter does not include significant variations at frequencies shorter than tens of days, which makes it directly more comparable with several-day LAIC processes and several-day changes in ionospheric signals. The sunspot numbers also exhibit unbroken increases within around 20-day windows, and therefore, both solar parameters are applied without averaging. The PCC of Swarm B in Figure 5a informs us about a notable correlation between Swarm signal and seismicity (PCCEQS = +0.44) and no positive correlation between Swarm and solar parameters (PCCF107 = −0.13, PCCSN = −0.24). In Figure 5b, respective correlations of Swarm are also slightly higher with seismic intensity (PCCEQS = +0.23, PCCF107 = −0.24, PCCSN = +0.13). In Figure 6a, representing Swarm C, the correlation with seismicity is also noticeable in PCCEQS = +0.11, but again negative in PCCF107 = PCCSN = −0.11. The year 2021 (Figure 6b) of higher solar activity reveals all Swarm C correlations at almost zero level (PCCEQS = −0.03, PCCF107 = +0.04, PCCSN = +0.05). A noticeable positive correlation of Swarm Ne increase with SN (Figure 5b) or comparable correlations of Swarm with seismicity and solar parameters (Figure 6b) can be also explainable, as several recent studies report on suspected relations between solar radiation and earthquakes [68,69]. The aim of this study is to initialize extraction of the frequencies, which are more sensitive to closer-distance processes acting from below than to more distant solar sources. Future spectral analyses in narrow spectral bands and with higher time–frequency resolution can help in more precise separation of the frequencies and identification of the signal sources. We believe that the presented spectrograms can be a starting point and helpful in the further planning of the next studies and data selection.

It must be pointed out that we calculated PCC values between ionospheric data, seismic intensity, sunspot number, and solar radio flux, assuming that the last three parameters represent single sources, i.e., the lithosphere or the Sun. We did not make PCC with geomagnetic indices. Instead, only a visual comparison with Dst is provided. The geomagnetic indices have the highest amplitudes of variations at wave periods significantly shorter than the 20-day window used for averaging of the Swarm data here, and their behavior is more dynamic than the changes studied here. The extreme peaks of Dst often coincide with individual large or moderate earthquakes. The relation of the geomagnetic field with earthquakes has been recently extensively studied [70,71], but it is also possible that the source of geomagnetic variations is twofold. Therefore, we rather focused on the comparison of ionospheric variations to drivers from above (Sun) and below (LAIC) than on the link between the seismicity and geomagnetic index, which obviously can have much in common.

The separation of earthquakes into six sequences in both years in Figure 5 and Figure 6 is based on their occurrence in close time and apparent separating time spans of lower seismicity. The Swarm Ne variations approximately occupy just the time spans of selected earthquake sequences but exhibit different power at different groups. Therefore, we prepared maps of these earthquake groups, illustrating their magnitudes and geographical distribution, which can facilitate a discussion on Ne disturbance power in relation to individual seismic sequence. It is worth noticing that some of these earthquake groups have broad geographical distribution, whereas some of them are located more locally, e.g., along the single plate boundary. Figure 7 presents the hypocenter and magnitude of the earthquakes that occurred in the short time intervals denoted by horizontal red lines. The intervals are numbered in Figure 5a and Figure 6a from s2020/1 to s2020/6, and Figure 7 presents earthquakes within these intervals together with orbital footprints of Swarm passes collected at respective times. The sequences s2020/1, s2020/3, s2020/5, and s2020/6 plotted in Figure 7a,c,e,f are located at different edges of the Hellenic Plate and therefore are widely distributed in many directions. The disturbances in Swarm Ne corresponding to these time spans are easily noticeable in Figure 5a and Figure 6a. The group s2020/2, in turn, has less extensive distribution but includes the earthquake with a larger magnitude of M = 5.7 (Figure 7b). The ionospheric Ne response at that time is large only in Figure 6a, for Swarm C crossing that region at a different time than Swarm B, which can suggest more transient disturbance. Figure 7d shows the earthquakes having small magnitudes up to M = 5.0 occurring during s2020/4. These earthquakes are located along one tectonic plate boundary and, at the same time, arranged in one geographical direction. A limited geographic range of s2020/4 series in combination with their low magnitudes are amongst the potential reasons of small responses in ionospheric Ne sensed by both Swarm satellites (Figure 5a and 6a).

The start of the year 2021 brought many earthquakes in the region (Figure 8a). The group of earthquakes in January (s2021/1) coincides with the largest Ne disturbances in the first half of 2021, recorded both by Swarm B (Figure 5b) and Swarm C (Figure 6b). The second sequence s2021/2 corresponds to smaller but also large ionospheric disturbances detected by Swarm B and relatively small disturbances found by Swarm C (Figure 8b). However, it can be suspected that the disturbances during s2021/1 and s2021/2 can be related to the same longer-term increased lithospheric activity, which is responsible for strongly disturbed Ne until the end of April 2021. The group s2021/3 seems to be affecting Swarm Ne the weakest of all groups. Figure 8c informs that the largest earthquake during s2021/3 was smaller (M = 5.3) than the largest in the preceding s2021/2 group. The first rough suspicion related to small disturbances in Swarm Ne at the time of s2021/3 can be such that 2021/3 occurred after s2021/2, and s2021/2 released most of the energy to the ionosphere. The fourth group s2021/4 brought for both Swarm satellites the largest disturbances, and a moderate M = 5.6 shallow earthquake was included in this group (Figure 8d). The magnitude of Ne disturbances at s2021/4 is larger in Figure 6b than in Figure 5b, and a variety of factors different between Swarm B and C can contribute to this fact, e.g., time between the earthquake and satellite pass or satellite altitude. However, these local characteristics are not studied here, as we focus on the occurrence of disturbances in Ne of both satellites undetected in 2020 at that time, and perfectly coinciding with M = 5.6 earthquake in the end of July 2021, after more sparse and weaker seismic activity in May and June. The end of the year 2021 is similar to the end of 2020, i.e., moderate earthquakes (s2021/6) seen in Figure 8f correlated in time with moderate Swarm B disturbance (Figure 5b) and large Swarm C signal variations (Figure 6b). The very dense-in-time earthquakes in group s2021/5 cause no significant disturbances of ionospheric Ne recorded by Swarm B and C. Figure 8e explains that these earthquakes, starting with the strong M = 6.0 earthquake, are very closely located in terms of hypocenter and have no correlated earthquakes at the opposite edges of the Aegean tectonic plate. Therefore, a small ionospheric response corresponding to the earthquakes in Figure 8e (s2021/5) can potentially have similar origin, as in the case shown in Figure 7d (s2020/4).

Summarizing, according to the descriptions related to short increased seismicity periods, the relatively calm months of seismic activity in both years coincide with the smallest disturbances of Swarm Ne, as observed, for example, in July 2020 or May 2021. The averaging of Swarm PSD maxima, as well as the calculation of average seismic intensity, is very helpful to support comparison studies, because the detected responses in the ionosphere are not exactly coinciding neither with the strongest earthquakes nor with the start of the series. The revisit time of Swarm satellites strongly impedes studies focused on particular seismic events. The variations of Ne at Swarm altitude coinciding with seismicity power change exhibit, in turn, a more long-term character of weeks, which, on the one hand, suggests the EFM channel of LAIC and, on the other hand, encourages temporal analysis of other long-term series of ionospheric observations, e.g., ground-based GNSS and Digisonde observations.

### 4.2. Variations of Ionospheric Characteristics from Athens Digisonde

The time series of the three selected ionospheric characteristics, foF2, foE, and ff, extracted from the ionograms of Athens Digisonde, require spectral filtering because strong solar activity affects some frequencies of the complex signal spectrum. Therefore, the spectrograms are calculated by STFT for high-pass filtered foF2, foEs, and ff data to recognize these parts of the signal, which are less disturbed by diurnal, semi-diurnal, and other wave periods sensitive to the solar activity (Figure 9, Figure 10 and Figure 11). The spectrograms of all three parameters indicate strong signals of diurnal and semi-diurnal wave periods, which dominate in the entire spectrum. These frequencies must be filtered out from the entire signal spectrum in order to analyze signals of much smaller amplitude, which can be sensitive to drivers other than the solar activity. After preliminary visual inspection of all spectrograms, it was decided to eliminate lower wave periods potentially correlated with solar activity like around the 27-day wave period. Then, having some review from the literature on EMF LAIC channel disturbances, we selected a preliminary experimental frequency band to test band-pass filter opportunities for Digisonde data. The frequency band indicating several PSD increases common with increased seismicity and visually less affected by the solar radiation was selected between 10 days and 6 days. This frequency band is filtered out from all three ionospheric characteristics and compared with the earthquakes’ records in 2020 (Figure 9b, Figure 10b and Figure 11b) and in 2021 (Figure 9d, Figure 10d and Figure 11d).

The band-pass filtered series of Digisonde-derived parameters are compared to seismic records and geomagnetic and solar indices, similarly to the maxima of sampled PSD in the case of Swarm. However, the band-pass filtered signal is an oscillating signal, and, in fact, the amplitude of variations determines the sensitivity of the selected signal band. Therefore, standard deviation of the selected signal band is calculated in 20-day time windows for the analysis of its correlation with geomagnetic and solar parameters. The standard deviation of band-pass filtered foF2, foE, and ff is presented using multiplied scales, adjusted to keep a similar magnitude within a single vertical axis with the other parameters (solar, geomagnetic, seismic) in Figure 9b,d, Figure 10b,d and Figure 11b,d.

The 20-day standard deviation of band-pass filtered time series of foF2, foEs, and ff shows notable coincidence with the same seismic intensity as in Section 4.1. The most pronounced coincidence of increased standard deviation of 6–10-day foF2 signal band and increased seismicity in 2020 in Figure 9b takes place from mid-September to mid-November. The standard deviation of the filtered foEs is very pronounced together with increased seismicity from the end of April until the end of June and also slightly increased in mid-October 2020 (Figure 10b). Figure 11b shows, over May and June, the maxima of standard deviation of filtered ff, which are consistent with seismic activity increase. There is also a noticeably higher signal in late October, when the largest M = 7.0 earthquake has occurred (Figure 11b). The analysis of Digisonde parameter coincidence with earthquake series in the end of January is impeded due to a large data gap in February–March 2020. There are also coincidences of the same characteristics with seismicity increase in 2021. Figure 9d reveals coherent increases in filtered foF2 standard deviation and seismic intensity in March and April, as well as in September 2021. In Figure 10d, the most evident coincidences can be found in September–October. Figure 11d presents, in turn, a continuous increase in standard deviation of band-pass filtered ff from January to May (decreased slightly between earthquake series in February), which converges with a seismically active time period at the same time. Summarizing, each of the three time spans of increased seismic activity in 2020, and two spans in 2021, have notable spatial correlation with at least one parameter from Athens Digisonde, but often with more than one.

Considering now two analyzed annual periods, both with three Ne parameters, the PCC values in five out of six cases are much higher in pairs of Digisonde–seismicity (PCCEQS) than in pairs of Digisonde–F10.7 (PCCF107) and in four out of six cases higher for PCCEQS in comparison to PCCSN. It must be pointed out that existing knowledge of LAIC processes acknowledges the high complexity of processes occurring in the ionosphere. Therefore, knowing the approximate character of PCC calculation, we did not expect high PCC values, as the signal at a wave period of 4–10 days can also be affected by the factors other than seismicity. However, we found that at the selected frequency band, we can observe signals potentially linked with seismic activity, and therefore, spectral analysis was assessed as worth implementation. The PCCEQS = −0.06, and there is a lower PCCF107 = −0.23 but close to zero PCCSN = +0.07 in 2020 in Figure 9b. The year 2020 has a significant data gap, affecting analysis, but aside from PCCs, we have found especially good correlation of foF2 with seismicity at a wave period of 4–10 days in October–November 2020 in Figure 9b. In 2021, the PCCEQS = +0.22, whereas PCCF107 = −0.03 and PCCSN = −0.09 in Figure 9d, and here we have a pronounced correlation with seismicity at least three times. The first time span of foF2 correlation with seismic intensity covers March and April 2021, the second one appears as precursory in September, and the third takes place at the end of December. The fourth increase at the beginning of June 2021 corresponds to a small increase in seismic energy at the May–June transition, which is also noticeable in Figure 6b, and Swarm C confirms Ne variation at that time. On the other hand, there is a small group of earthquakes, and the reason for lower seismic energy could stem from the large depth of the earthquake at the beginning of June 2021 included in its calculation. The PCCEQS = +0.47, whereas PCCF107 = −0.19 and PCCSN = −0.13 in Figure 10b, and this is a relatively good correlation of foEs with seismicity in 2020. Nevertheless, for 2021, summarized in Figure 10d, we have the PCCEQS = −0.30, PCCF107 = −0.09, and PCCSN = −0.12. This is the only case when the PCC of the Digisonde-derived parameter with seismicity is lower than with solar parameters. Despite these PCC values, we have there a good Digisonde–earthquakes coincidence in September, but there are also two increases in filtered foEs standard deviation in May and June, having two following F10.7 increases several days later (Figure 10d). This anomaly can have a common source with those in Figure 6b and Figure 9d, where a small group of earthquakes includes one of large depth, which can contribute to decreased calculated seismic intensity. The deeper analysis of the relation between Digisonde-derived ionospheric parameters, seismicity, and solar activity needs more local study with more resolution in the frequency domain, which can be better selected after initial observations from the current analysis. Figure 11 presents notable positive correlations of the ff parameter with seismicity and low negative with the Sun. In 2020, the PCCEQS = +0.17, but PCCF107 = −0.56 and PCCSN = −0.02 in Figure 11b. Similarly, in 2021, PCCEQS = +0.33, but PCCF107 = −0.41 and PCCSN = −0.24 in Figure 11d. Summarizing, these six comparisons encourage more detailed spectral analysis, within limited frequency bands and increased time–frequency resolution, which exceeds the scope and reasonable page limit of this study.

### 4.3. Variations of VTEC from UQRG

The UQRG-based VTEC has 15 min. temporal resolution, which is also well sufficient for the analysis of periodic signals longer than diurnal. Therefore, two years of VTEC time series interpolated at the fixed position (38° N, 24° E) near Athens are detrended and processed in the same way as the selected Digisonde-extracted characteristics. The spectrogram is divided similarly into two parts, 2020 and 2021, and both parts have very pronounced diurnal and semi-diurnal components, which are even more evident in the entire spectrum in comparison to Digisonde-based characteristics. In turn, we can see in VTEC spectrograms a higher sensitivity to solar radio flux at lower frequencies, which is especially pronounced in 2021, when the F10.7 parameter varies in the second half of the year (Figure 12c,d). The previously discussed cumulative character of TEC can contribute to this fact. In order to be consistent with Digisonde data analysis, we set the bounds for band-pass filtering also at the 6–10 days level for experimental correlation analysis of filtered annual VTEC series. The band-pass filtered signal is an oscillating signal, as in the case of Digisonde characteristics, and again, the 20-day standard deviation of the selected signal band is applied in the analysis of its correlation with geomagnetic and solar parameters.

The standard deviation of band-pass filtered VTEC is also shown using a multiplied scale to be presentable with respect to the other parameters in the single vertical axis, similarly to the Digisonde case in Section 4.2. There are three time intervals of stronger seismicity in the Aegean Sea in 2020: February, May–June period, and the second half of September with October (Figure 12b). The standard deviation of band-pass filtered VTEC increases clearly during the two first and slightly before the third one. It should be mentioned that GIM VTEC comes from kriging interpolation of signals between many stations and satellites, which contributes to spatial signal smoothing. There is a single peak of VTEC at the end of July 2020, which coincides with two shallow earthquakes, but also with magnetospheric activity seen in the Dst index. In 2020, the PCCEQS = +0.16, whereas PCCF107 = +0.22 and PCCSN = −0.13 in Figure 12b, which informs us about a similar level of correlation with seismicity and solar activity if we take the F10.7 parameter into account. We must keep in mind that the GNSS signals are integrated Ne values up to 20,000 km, and many altitudes can be affected more by the Sun than in the case of ionosonde signal.

There are also two longer time intervals of the strongest seismic activity in 2021: January–mid-April and mid-July to the end of October (Figure 12d). The standard deviation of filtered VTEC increases very clearly in both periods, also in the time of calm solar radio flux. Moreover, the behavior of VTEC in 2021 shown in Figure 12d is very similar to variations of the ff parameter in Figure 11d. The PCCEQS = +0.29, whereas PCCF107 = −0.26 and PCCSN = −0.11 in Figure 12d, which gives advantage to the earthquakes. Summarizing, the standard deviation of filtered VTEC averaged in 20-day windows corresponds to events of increased seismicity much more than to the solar radio flux increase, or to the largest increases in sunspot number. These results again need more resolution in time–frequency in future research.

## 5. Conclusions

This study presents STFT analysis of two-year time series of five ionospheric parameters from the satellites and from the ground-based instrumentation. These are in situ LEO Ne observations in the topside ionosphere, Digisonde-derived characteristics in the Es and the F layer in the bottom-side ionosphere (foF2, foEs, ff), and the VTEC from the UQRG GIM. This study demonstrated a promising applicability of band-pass filtering in the analyses of correlation between ionospheric parameters and seismicity. The spectrograms revealed the complexity of the signals, a strong drawback of unfiltered signals coming from diurnal and seasonal components, and an interesting potential sensitivity of relatively weak several-day signals to seismicity. The spectrograms of Swarm along-track Ne allowed for the selection of Swarm PSD from the experimental 35-s wave period and creation of time series. The Fourier-based band-pass filter with experimental bounds of 6–10 days separated signal parts from Digisonde and GIM VTEC time series. Despite the partially intuitive selection of wavebands based on visual analysis at this stage of research, it was demonstrated that ionospheric variations at these bands coincide with increased seismicity in the Aegean region numerous times. The PCC coefficients are treated relatively and with caution in this work and only support visual inspections, which provide a full view of the number of coincident signals in the analyzed years. The PCC values between selected ionospheric signals and seismicity (PCCEQS) were not expected to reach high values due to the variety of factors affecting the signals and incomplete knowledge on precursory and post-seismic signals in LAIC. Nevertheless, the PCCEQS values are within 0.2–0.5 in the predominant number of cases, which is noteworthy. Similar PCC values calculated with the F10.7 parameter (PCCF107) and the SN parameter (PCCSN) are negative in most of the cases. Counting this in a different way, the PCCEQS is higher than both PCCF107 and PCCSN in 8 out of 12 comparisons. This notifies us that selected wavebands are candidates to be related to seismicity.

The suspicion of longer-term correlations of seismicity with high PSD of selected Swarm Ne signal band, a selected signal band of bottom-side ionospheric time series from Digisonde, and time series of VTEC from UQRG is based on the significant number of resonant extrema and a notable number of positive PCC values. The EFM channel of LAIC is the most strongly suspected of triggering detected ionospheric variations, mainly due to its longer-term character with respect to AGW, and this is in compliance with [16]. Regarding the Digisonde-derived data and VTEC data, it must be pointed out that these observations come from long ionospheric profiles contrary to Swarm in situ Ne. Therefore, foF2, foEs, ff, and VTEC signals are more affected by various phenomena at different heights and thus more complex and more challenging in respect of band-pass filtering. Therefore, at least stationary ionospheric time series need much more detailed spectral analysis in the future.

This demonstrative STFT application assured the detection of relatively weak signals, which reveal more correlation with seismic records than with sunspot number and solar radio flux There are, however, limitations of this study: unknown shift between seismic and ionospheric events that can be different for different seismic events, potential relation between solar parameters and seismicity that is often recently reported, and too low time–frequency resolution of spectral analysis at this step. Reprocessing with more time–frequency resolution in limited wavebands is required for more accurate waveband selection, but this needs more additional plots and their analysis. Nevertheless, the number of notable correlations of ionospheric data with seismicity encourages the continuation of the work, especially if we have at our disposal decades of data having several-minute resolution.

## Data Availability

Swarm data are downloaded from the ESA Data Access Service (https://swarm-diss.eo.esa.int accessed on 2 December 2024, ftp://swarm-diss.eo.esa.int accessed on 2 December 2024) The earthquake series are downloaded from the United States Geological Survey service (https://earthquake.usgs.gov/earthquakes/search/ accessed on 2 December 2024). Sunspot data are from the World Data Center SILSO [72], Royal Observatory of Belgium, Brussels. The Solar Radio Flux data are downloaded from the website of International Reference Ionosphere (IRI) of the Committee on Space Research (COSPAR) (http://irimodel.org/ accessed on 2 December 2024). The results presented in this paper rely on geomagnetic indices calculated and made available by ISGI Collaborating Institutes from data collected at magnetic observatories. We thank the involved national institutes, the INTERMAGNET network and ISGI (https://isgi.unistra.fr accessed on 2 December 2024). Tectonic plate boundaries are downloaded from data webpage: (https://github.com/lcx366/PlateTectonic accessed on 2 December 2024).

## Abbreviations

AGW	acoustic gravity waves;
DFT	discrete Fourier transform;
EFM	electric field modifications;
ff	frequency spread;
foEs	maximum frequency of the sporadic E layer;
GIM	global ionospheric map;
GNSS	global navigation satellite system;
foF2	maximum frequency of the F2 layer;
LAIC	lithosphere–atmosphere–ionosphere coupling;
LEO	low-Earth orbiting (satellite);
LP	Langmuir probe;
Ne	electron density;
NOA	National Observatory of Athens;
PSD	power spectral density;
SN	sunspot number;
STFT	short-term Fourier transform;
TEC	total electron content;
UQRG	UPC-IonSAT quarter-of-an-hour time resolution rapid global ionospheric map;
USGS	United States Geological Survey;
VTEC	vertical TEC.
